# Testosterone affects type I/type II interferon response of neutrophils during hepatic amebiasis

**DOI:** 10.3389/fimmu.2023.1279245

**Published:** 2023-12-21

**Authors:** Marco Er-Lukowiak, Sonja Hänzelmann, Moritz Rothe, David T. Moamenpour, Fabian Hausmann, Robin Khatri, Charlotte Hansen, Jennifer Boldt, Valentin A. Bärreiter, Barbara Honecker, Annika Bea, Marie Groneberg, Helena Fehling, Claudia Marggraff, Dániel Cadar, Stefan Bonn, Julie Sellau, Hanna Lotter

**Affiliations:** ^1^ Molecular Parasitology and Immunology, Bernhard Nocht Institute for Tropical Medicine, Hamburg, Germany; ^2^ Institute of Medical Systems Biology, University Medical Center Hamburg-Eppendorf, Hamburg, Germany; ^3^ Center for Biomedical Artificial Intelligenc, University Medical Center Hamburg-Eppendorf, Hamburg, Germany; ^4^ III. Department of Medicine, University Medical Center Hamburg-Eppendorf, Hamburg, Germany; ^5^ Hamburg Center for Translational Immunology, University Medical Center Hamburg-Eppendorf, Hamburg, Germany

**Keywords:** sex difference, Entamoeba histolytica, testosterone, neutrophils, type I and type II interferon

## Abstract

Differences in immune response between men and women may influence the outcome of infectious diseases. Intestinal infection with Entamoeba histolytica leads to hepatic amebiasis, which is more common in males. Previously, we reported that innate immune cells contribute to liver damage in males in the murine model for hepatic amebiasis. Here, we focused on the influences of sex and androgens on neutrophils in particular. Infection associated with neutrophil accumulation in the liver was higher in male than in female mice and further increased after testosterone treatment in both sexes. Compared with female neutrophils, male neutrophils exhibit a more immature and less activated status, as evidenced by a lower proinflammatory N1-like phenotype and deconvolution, decreased gene expression of type I and type II interferon stimulated genes (ISGs) as well as downregulation of signaling pathways related to neutrophil activation. Neutrophils from females showed higher protein expression of the type I ISG viperin/RSAD2 during infection, which decreased by testosterone substitution. Moreover, ex vivo stimulation of human neutrophils revealed lower production of RSAD2 in neutrophils from men compared with women. These findings indicate that sex-specific effects on neutrophil physiology associated with maturation and type I IFN responsiveness might be important in the outcome of hepatic amebiasis.

## Introduction

Sex chromosomes, hormones, and the immune system influence disease severity and susceptibility in men and women (1–3). Male hormones can suppress the immune system, making men more vulnerable to infections, while female hormones promote stronger immune responses, albeit with a potential for autoimmune diseases (4). However, precise mechanisms underlying these differences remain unclear.

Innate immune cells (neutrophils and monocytes) are crucial for the initial response against microorganisms. However, inadequate control can lead to their involvement in the pathophysiology of different diseases (5, 6). Neutrophils are the most abundant immune cells and are recruited to affected tissues in large numbers (7–10). Neutrophils are traditionally considered a homogeneous population expressing CD11b, Ly6C, and Ly6G in mice and CD66, and CD16 in humans (11–13). However, human and murine single cell RNA studies show that neutrophils are a more heterogeneous immune cell population (14–17). By analogy with the classification of macrophages into proinflammatory M1-like and anti-inflammatory M2-like cells (18), neutrophils in mice in the tumor environment had been classified as proinflammatory (N1) and anti-inflammatory (N2) based on expression of specific surface markers (CD54 and PDL-1, respectively (19, 20). However, it is unclear whether this classification can be applied in the context of infectious diseases. In the mouse model for hepatic amebiasis caused by infection with the parasite *Entamoeba histolytica* (*E. histolytica*), which is more common in males than in females, neutrophils contribute to the development of liver injury (21–23). Testosterone treatment or gonadectomy reverse these sex-related differences, and depletion of neutrophils or their recruitment factor (CXCL1) reduces liver destruction significantly (23–25). Female mice, on the other hand, are protected by strong amoebicidal interferon (IFN)-γ responses (22, 24, 26, 27). Interferons are a broad family of molecules categorized as type I (IFN-α/β), type II (IFN-γ), and type III (IFN-Λ1-4) (28). The corresponding signaling pathways induce the expression of the respective interferon-stimulated genes (ISGs) (29–31) Type I IFN production and those of the corresponding ISGs, including the interferon-inducible virus-inhibitory protein (viperin) encoded by Rsad2, are increased at the RNA level in neutrophils from women compared with men, however, no sex difference in their number has been reported (16). This is significant as higher viperin/RSAD2 expression is often associated with more effective viral elimination by targeting virus proteins for proteasomal degradation (32, 33) and catalyzation of ribonucleotides that inhibit viral RNA synthesis (34). However, in non-viral infectious diseases, not much is known about the function of ISGs.

Here, we examined sex-specific involvement of neutrophils in the murine model of hepatic amebiasis. Male mice had higher testosterone-dependent neutrophil presence compared to females. Testosterone reduced infection-related N1 and N2-like neutrophils, along with an increase in maturation stage in female neutrophils. Female neutrophils showed higher gene expression of type I and type II ISGs compared to male neutrophils, with viperin/RSAD2 being upregulated in neutrophils at the protein level of female mice and women and downregulated after testosterone treatment of mice.

## Materials and methods

### Human samples

All human studies complied with all relevant ethical regulations. Experiments with blood samples from healthy caucasian donors of both sexes (25–49 years of age) were approved by the ethics committee of the medical association Hamburg (permission number: 2020-10067-BO). All experiments were conducted under donor anonymization and in accordance with the relevant guidelines.

### Animal experiments

C57BL/6 mice (10–12 weeks old) were used for the experiments, which were performed in accordance with German animal protection laws and reviewed by the federal health authorities of the State of Hamburg (permission numbers: N51/17; N120/2020). Mice were bred in the animal facility of the Bernhard Nocht Institute for Tropical Medicine and kept at 21–22°C (50–60% humidity) in ventilated cages under specific pathogen-free conditions. Mice were rendered unconscious using a CO_2_ (20%)-filled chamber and sacrificed by cervical dislocation.

### Induction of hepatic amebiasis

Mice (10-12 weeks old) were infected intrahepatically with 2 × 10^5^ *in vitro*-cultivated trophozoites of the high pathogenic *E. histolytica* clone B2, as described previously (35, 36). Mice were sacrificed on day 3 postinfection (pi), at the peak of disease severity (25).

### Gonadectomy, and testosterone treatment of male and female mice

Male mice (8 weeks old) were gonadectomized by testicular ligation. Testosterone substitution was performed by subcutaneous implantation of an osmotic pump (micro-osmotic pump; Model 2004, ALZET) containing 5 mg/mL testosterone diluted in 45% w/w 2-Hydroxypropyl-β-cyclodextrin (carrier solution) or carrier solution alone. Alternatively, testosterone implants (Belma Technologies: T30) were inserted subcutaneously.

### Isolation of murine immune cells

Bone marrow (BM) was collected from euthanized mice by flushing it into a dish using PBS. The cell suspension was filtered and treated with an erythrocyte lysis buffer. Blood was obtained by cardiac puncture upon euthanization, collected in EDTA-coated tubes, and two erythrolysis steps were performed to isolate immune cells. Spleen and liver were mashed, flushed with PBS on ice, and filtered, followed by one erythrolysis step. Liver immune cells were isolated using 80% Percoll as described previously (37). Neutrophil granulocytes from bone marrow were isolated using a Neutrophil Isolation Kit (#130-097-658, Miltenyi Biotec), blood neutrophils were isolated by FACS-sorting.

### Histology and immunohistochemistry

Liver tissue from *E. histolytica*-infected mice was fixed in formalin (4%) and embedded in paraffin. Sections (0.2 µm) were stained with hematoxylin and eosin (H&E) or prepared for immunohistochemistry. Antibodies: rabbit anti-mouse 7/4 antibody (Neutrophils, clone 7/4; Cedarlane; 1:800 dilution); polyclonal rabbit serum raised against recombinant *E. histolytica-* antigens. Slices were developed using DCS SuperVision Single Species horse-radish peroxidase (HRP)-Polymere (Innovative Diagnostic-Systems) and counterstained with hemalaun.

### Cytokine analysis

Blood plasma was used for cytokine analysis. Briefly, collected blood was centrifuged (1000 × g, 4°C, 10 min) to obtain plasma and stored -20°C prior to cytokine measurement. Cytokine analysis was performed using multiple customized murine LEGENDplex kits (BioLegend).

### Flow cytometry analysis

Flow cytometry was performed from organ single-cell suspensions or purified neutrophils, live cells were identified using zombie UV dye (#423108 BioLegend) or live/dead blue (#L34961 Invitrogen), intracellular cytokine staining was performed following 4 h restimulation with phorbol myristate acetate (PMA; 50 ng/mL) and ionomycin (500 ng/mL), followed by a 1h Brefeldin A (5 µg/ml) incubation. Antibodies: (all from BioLegend unless stated otherwise): CD11b FITC (1:200, M1/70), CD11b PerCP (1:100 M1/70), CD11b Bv510 (1:100, M1/70), Ly6C PerCP/Cy5.5 (1:400, HK 1-4), Ly6C PE (1:800, HK 1-4), Ly6G APC/Cy7 (1:400, 1A8), Ly6G PE (1:400, 1A8), Ly6G Bv785 (1:100, 1 A8), PD-L1 PE (1:100, 10F.9G2), CD54 AF647 (1:200, YN1/1.74), CD117 BUV395 (1:100, 2B8, BD), TNF-α BV421 (1:200, MP6-XT22), CCL2 PE (1:50, 2H5), CXCL1 AF647 (1:50, 1174A, R&D). Human neutrophils were analyzed in peripheral blood or after isolation using the MACSxpress^®^ kit (Miltenyi Biotech). Isolated cells were stimulated with PMA (10 ng/mL), LPS (0.1 µl/mL) and CL097 (1µg/mL) for 4 hours. Antibodies: CD16-APC-Cy7 (1:200, 3G8) and CD66B-APC (1:400, G10F5, Biolegend), viperin (RSAD2) PE (1:200, MaP,VIP; Biosience). Flow cytometry analysis was performed on a Cytek Aurora (Cytek)- or BD LSRII cytometer and data analysis were performed using FlowJo V10.4.2 software.

### RT-PCR/qPCR

RNA was isolated from BM-derived neutrophils or blood leukocytes using TRIzol reagent (Life Technology) or a RNeasy MinElute kit (Qiagen). RNA was transcribed into cDNA using the Maxima First Strand cDNA Kit (Thermo Scientific). Androgen receptor (AR) and Ly6G mRNA levels were calculated using the 2^-ΔΔCt^ method, with the ribosomal protein S9 (RPS9) as housekeeping gene. The following primers were used: fwd-AR, 5´-TGAGTACCGCATGCA-CAAGT-3´; rev-AR, 5´-GCCCATCCACTGGAATAATGC-3´. Ly6G SG QuantiTect primers (Qiagen) were used for quantification of Ly6G. QPCR was performed using the Maxima SYBR Green qPCR Master Mix kit (Thermo Scientific) and a Roche LightCycler^®^.

### RNA sequencing and data analysis

Sequencing library was prepared using the QIAseq Stranded mRNA Library Kit (Qiagen, Hilden, Germany) at the NGS Core Facility at the Bernhard-Nocht-Institute for Tropical Medicine and sequenced on a NextSeq 2000 Illumina Platform. Constructed libraries were sequenced as barcoded pooled samples on a NextSeq 550, resulting in 14–17.5 million reads for blood and 15.1–22.4 million reads for BM. The read length of the nucleotides was 100 base pairs. Library preparation and sequencing were performed at the Bernhard Nocht Institute for Tropical Medicine.

### Bioinformatics

#### Cell type deconvolution analysis

Single cell reference-based cell-type deconvolution was performed using blood and BM RNA-seq samples based on the neutrophils single-cell data generated by Kim et al. (38) which uses the nomenclature introduced in Xie et al. (17), as the reference for deconvolution by Scaden (39). The single cells (n=6025) were labeled G2 (n=67), G3 (n=1495), G4 (n=2843), G5a(n=1235), G5b (n=92) and G5c (n=293). Scaden uses a fully connected deep neural network ensemble trained on pseudobulks simulated from reference scRNA-seq data. Before deconvolution, scRNA-seq data were filtered using scanpy.pp.filter cell function from Scanpy (40) with arguments min counts=200 and min genes=5. For Scaden, counts per million (CPM) of simulated pseudobulks and transcripts per million (TPMs) of bulk RNA-Seq to be deconvolved, were used.

#### Quantification

To compare murine samples, transcript counts were normalized using DESeq2 size factor estimation. Subtype-specific differential expression of transcripts was determined using a 2-fold change cut-off and an adjusted *p*-value <0.05 (unless stated otherwise). Sex-specific differences were analyzed from BM and blood neutrophils in steady state or following infection. In addition, blood and BM neutrophils from ALA versus WT males and females were analyzed separately to examine nonsex-related effects of the disease.

#### Differential exon usage

Alternative isoform usage was detected using Nextflow nf-core/rnasplice (v1.0dev) and DEXSeq (version 1.36.0 under R version 4.0.3, using exon annotation from Ensembl version GRCm38), which analyze exon-by-exon changes in expression based on RNAseq data (41). A 1.5- fold change (FC) cut-off and adjusted *p*-value <0.05 were used to determine significance.

#### Transcription factor inference

The Python version of DecoupleR (42) was used for TF activity estimation, which was performed using the DoRothEA database (43). TF-target interaction pairs were filtered from DoRothEA according to the confidence level of the annotation, and the three highest confidence levels were retained to create a predictive model for TF activity. The consensus estimates from weighted sums (wsum), univariate linear models (ulm), and multivariate linear models were used to estimate TF activity in each sample.

#### Clustering

Following removal of sex-dependent genes by differential expression analyses, the raw count matrix was subject to a variance stabilizing transformation (44) to address heteroskedasticity in gene counts. Heatmaps were generated using Seaborn in Python (version 3.9.7).

#### Enrichment analysis

Enrichment of pathway activation signatures was analyzed using the Python package gseapy (45) (v1.0.3) along with the prerank function of the gene ontology (GO) biological processes, KEGG and MSigDB databases.

#### Statistical analysis

All data were analyzed with the Shapiro-Wilk test for normal distribution prior testing for statistical differences between groups. Statistical analysis is indicated in each figure legend and was carried out using either an unpaired student’s t test for normal distribution. For non-normal distribution, a nonparametric Mann-Whitney U test was used (GraphPad Prism V.9). Bonferroni-Dunn correction was performed to account for multiple testing. P-values are indicated as *p< 0,05, **p< 0,01, ***p< 0,001.

## Results

### Sex-specific differences in neutrophils and cytokine production during hepatic amebiasis

**Figure 1:**
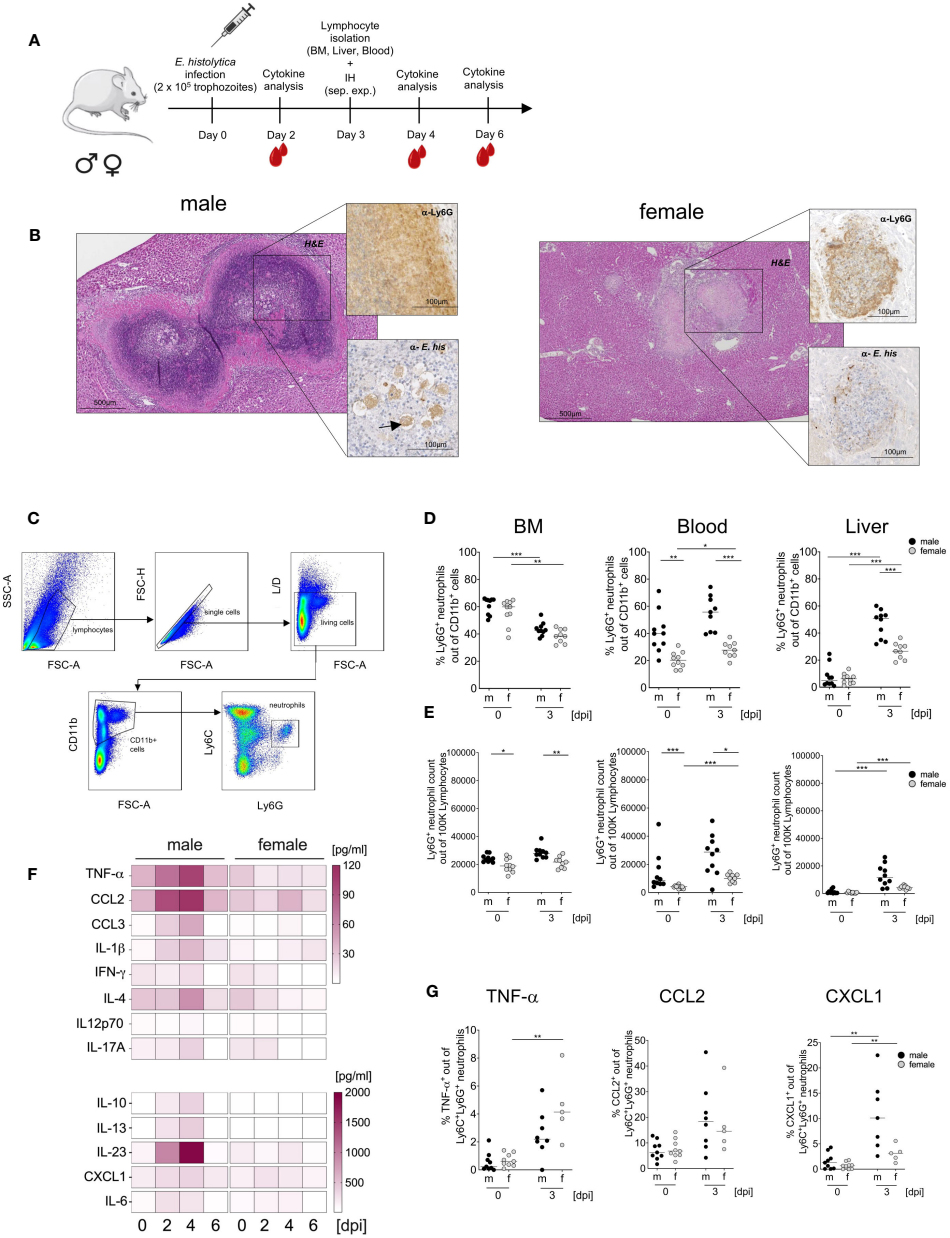
Sex-specific differences in neutrophil recruitment and cytokine production during hepatic amebiasis. **(A)** Illustration showing induction of amoebic liver abscess (ALA). **(B)** Immunohistochemical analysis of paraffin-embedded livers from male and female mice at day 3 post infection (H&E: Hematoxylin & Eosin; α-Ly6G: 7/4 mAb; α-*E. histolytica*: polyclonal serum). **(C)** Flow cytometry gating strategy used to identify neutrophils (CD11b^+^Ly6C^+^Ly6G^+^) in murine bone marrow (BM), blood, and liver. **(D)** Percentage and **(E)** relative number of Ly6G^+^ neutrophils in BM, blood, and liver of male (m) and female (f) mice before and at day 3 postinduction of ALA. **(F)** Serum cytokine levels (pg/ml) in female and male mice during hepatic amebiasis (days post infection [dpi]), as measured by a bead-based immunoassay (BioLegend) (n = 6–8/sex). **(G)** Percentage of TNF-α-, CCL2-, and CXCL1-positive neutrophils in the liver of male (m) and female (f) mice before and after ALA induction, as measured by flow cytometry. (**D**-Blood) (**E**-BM) (**G**-CCL2, CXCL1) p-values were calculated using a two-tailed Student’s t test. (**D**-BM, Liver) (**E**-Blood, Liver) (**G**- TNF-α) p-values were calculated using a two-tailed Mann-Whitney-U test. (*p < 0.05, **p < 0.01, ***p < 0.001).

As previously shown, depletion of inflammatory Ly6C^hi^ monocytes and neutrophils reduces liver damage in the murine model for hepatic amebiasis (23, 25). The same experimental design was used to study neutrophil granulocytes: Male and female mice were infected intrahepatically with *E. histolytica* trophozoites and examined on day 0 and day 3 post infection. (**Figure 1A**). Immunohistological examination of infected livers demonstrated greater tissue damage, massive immune cell infiltration, stronger abscess formation and a distinct margin area of Ly6G^+^ neutrophils in males than in females (**Figure 1B**). Flow cytometry analysis identified neutrophils as Ly6G^+^ cells out of CD11b^+^ cells (**Figure 1C**) and revealed higher percentages of neutrophils in the blood of naive male animals, but not in the BM and liver; after infection, a significant increase in the amounts of neutrophils in the blood of female animals and in the liver of both sexes was observed (**Figures 1D, E**).

The more severe pathology in males was reflected by higher pro- and anti-inflammatory cytokine serum levels in male than in female mice (**Figure 1F**). Infection triggered an increase in neutrophil accumulation in the liver; these cells produced TNF-α (significantly higher in females after infection), CCL2, and CXCL1, and the percentage of neutrophils producing CXCL1 was significant higher in males and females compared to uninfected mice (**Figure 1G**). In addition, phagocytic capacity (**Supplementary Figures 1A, B**), production of reactive oxygen species (ROS) (**Supplementary Figures 1C, D**) as well as myeloperoxidase (MPO) production (**Supplementary Figures 1E, F**) as assayed on BM-derived neutrophils, was not significant higher in female neutrophils than in male counterparts (**Supplementary Figure 1A**). In summary, amoebic infection of the liver results in a higher prevalence of neutrophilic granulocytes in male mice.

### Effect of testosterone on neutrophil accumulation and maturation during hepatic amebiasis

**Figure 2:**
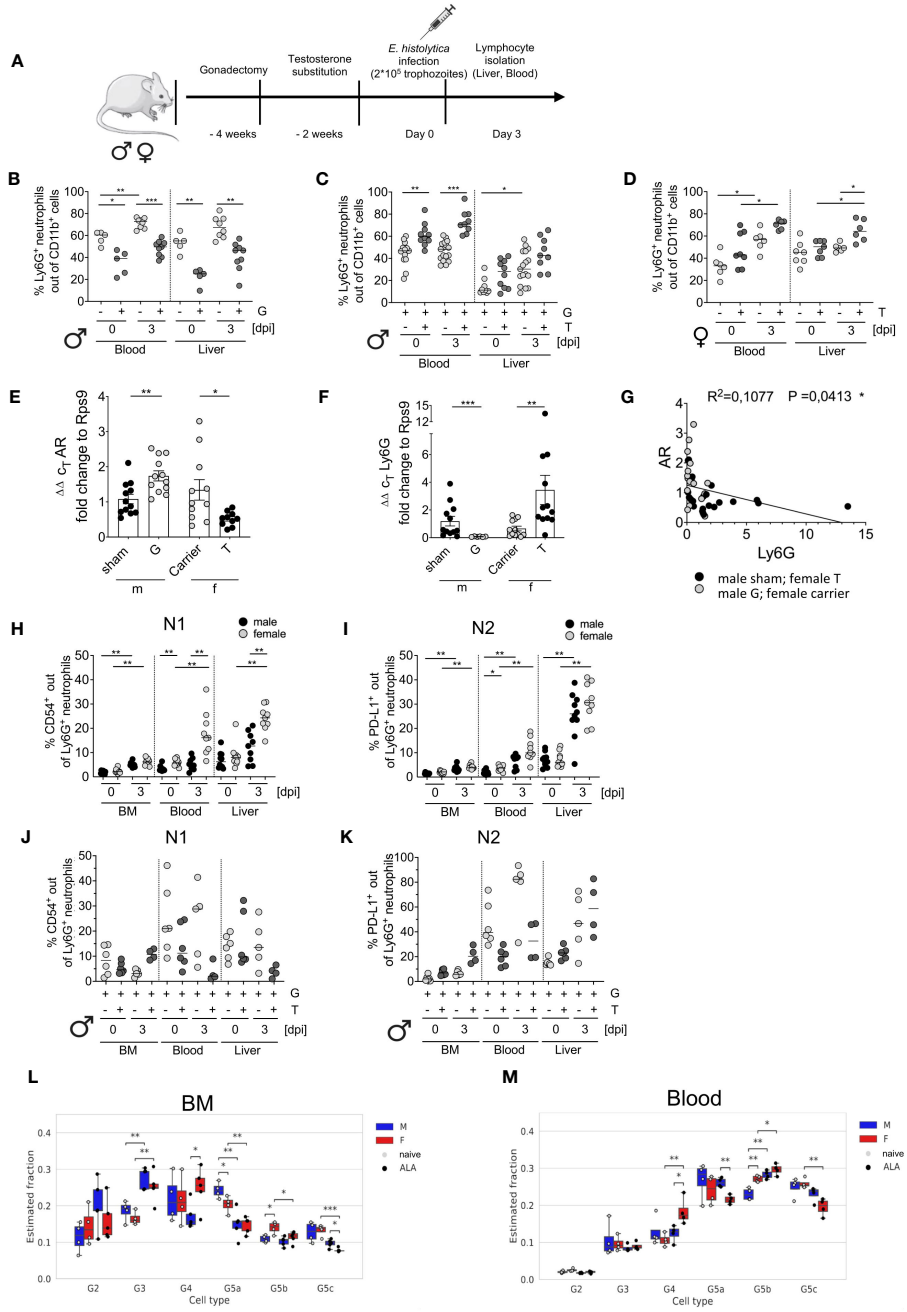
Impact of gonadectomy and testosterone substitution on neutrophil recruitment and phenotype characteristics during ALA. **(A)** Illustration of the testosterone substitution study. **(B)** Percentage of neutrophils (CD11b^+^Ly6C^+^Ly6G^+^) in the blood and liver of gonadectomized (G) male mice before and after (0 and 3 days post infection [dpi]) with *E*. *histolytica*, as measured by flow cytometry. **(C)** Percentage of neutrophils in the blood and liver of G and testosterone-substituted (T) male mice at 0 and 3 dpi with *E*. *histolytica*, as measured by flow cytometry. **(D)** Percentage of neutrophils in the blood and liver of T female mice at 0 and 3 dpi, as measured by flow cytometry. Expression of mRNA encoding **(E)** androgen receptor (AR1) and **(F)** Ly6G by blood leukocytes from naïve, sham-operated, or castrated male mice, and female mice, treated with carrier solution or testosterone, as measured by RT-qPCR. **(G)** Correlation between AR and Ly6G mRNA expression by isolated murine blood cells. Percentage of N1 **(H)** and N2 **(I)** neutrophils in the BM, blood, and liver of males and females on day 3 post infection with *E*. *histolytica.* Percentages of N1 **(J)** and N2 **(K)** neutrophils following male gonadectomy (G) and testosterone (T) substitution. **(L, M)**. Deconvolution analysis showing the neutrophil maturity stage (G2, G3, G4, G5a, G5b and G5c) of male and female mice in naïve- or ALA conditions. Analysis was performed for neutrophils derived from bone marrow **(K)** and blood **(L)**. **(B, D, E, I)** p-values were calculated using a two-tailed Student’s t test **(C, F, H, J, K)** p-values were calculated using a two-tailed Mann-Whitney-U test (*p < 0.05, **p < 0.01, ***p < 0.001). **(G)** A two-tailed Pearson’s correlation coefficient with simple linear regression (*p < 0.05).

To study the impact of testosterone on neutrophil dynamics during *E. histolytica* infection, male mice underwent gonadectomy and then received either testosterone or a carrier solution for two more weeks before being infected intrahepatic with *E. histolytica* (**Figure 2A**). Gonadectomy of male mice significantly reduced the number of neutrophils, regardless of infection (**Figure 2B**). In contrast, testosterone substitution in gonadectomized naïve male mice resulted in a marked increase in neutrophil abundance in the blood and tended to increase in the liver as well. (**Figure 2C**). A similar effect on neutrophils was observed in female individuals substituted with testosterone (**Figure 2D**). qPCR analysis of blood leukocytes revealed increased AR and reduced Ly6G mRNA expression in males following gonadectomy, whereas testosterone treatment reduced expression of AR mRNA and increased expression of Ly6G mRNA in females (**Figures 2E, F**). Consequently, there was a correlation between expression of both, AR and Ly6G (R^2 = ^0,1077; *p* < 0.05) (**Figure 2G**). Further, sex-specific correlation analyses are provided in **Supplementary Figures 2C, D**. Next, we analyzed the expression of CD54^+^ (inflammatory N1) and PD-L1^+^ (anti-inflammatory N2) on neutrophils (CD11b^+^Ly6C^+^Ly6G^+^) (scheme and representative FACS Plots are shown in **Supplementary Figures 2A, B**). The amount of N1 neutrophils increased after infection in the BM, blood and liver in female individuals, as well as in the BM and the liver in males (**Figure 2H**). N2 neutrophils showed a similar pattern as N1, with the greatest quantities in the liver in both sexes (**Figure 2I**). No significant differences were found in N1 neutrophils following gonadectomy and testosterone substitution. Only a slight decrease in the BM following infection as well as in the blood and the liver following testosterone substitution and infection was observed (**Figure 2J**). Although not significant, N2 neutrophils are increased in the blood in naive gonadectomized and infected male mice upon testosterone treatment, and increased in gonadectomized and testosterone substituted mice in the liver upon infection (**Figure 2K**).

*Ex vivo* exposure of isolated BM-derived neutrophils increased expression of CD54 (N1) and PD-L1 (N2) following LPS stimulation in both sexes and increased production of TNF-α in males following stimulation with amoebic antigens (**Supplementary Figures 3A–C**).

Based on the data published by Kim et al. (38) and the nomenclature of Xie et al. (17), deconvolution analysis was performed to investigate further maturation stages. Pre-neutrophils (G2) were present in the BM but not in the blood. Immature neutrophils (G3) increased after infection. Mature neutrophils (G4) and ISG-expressing neutrophils (G5b (17, 38);) significantly increased in infected females’ BM and blood. Late-mature neutrophils (G5a, G5c; migration & inflammatory responses (17, 38);) decreased in the BM and in the blood in females during infection but increased in males after infection (**Figures 2L, M**).

In summary, not all neutrophil granulocytes meet the N1- and N2-like definitions and testosterone further suppresses both of them in the blood during infection. Females tend to have a higher proportion of N1 and N2 neutrophils in the blood and liver, and deconvolution analysis revealed that a higher number of mature neutrophils (G4) is found in females and G5a neutrophils are higher in males during *E. histolytica* infection in the blood.

### Overall gene expression is different in BM neutrophils and blood neutrophils

**Figure 3:**
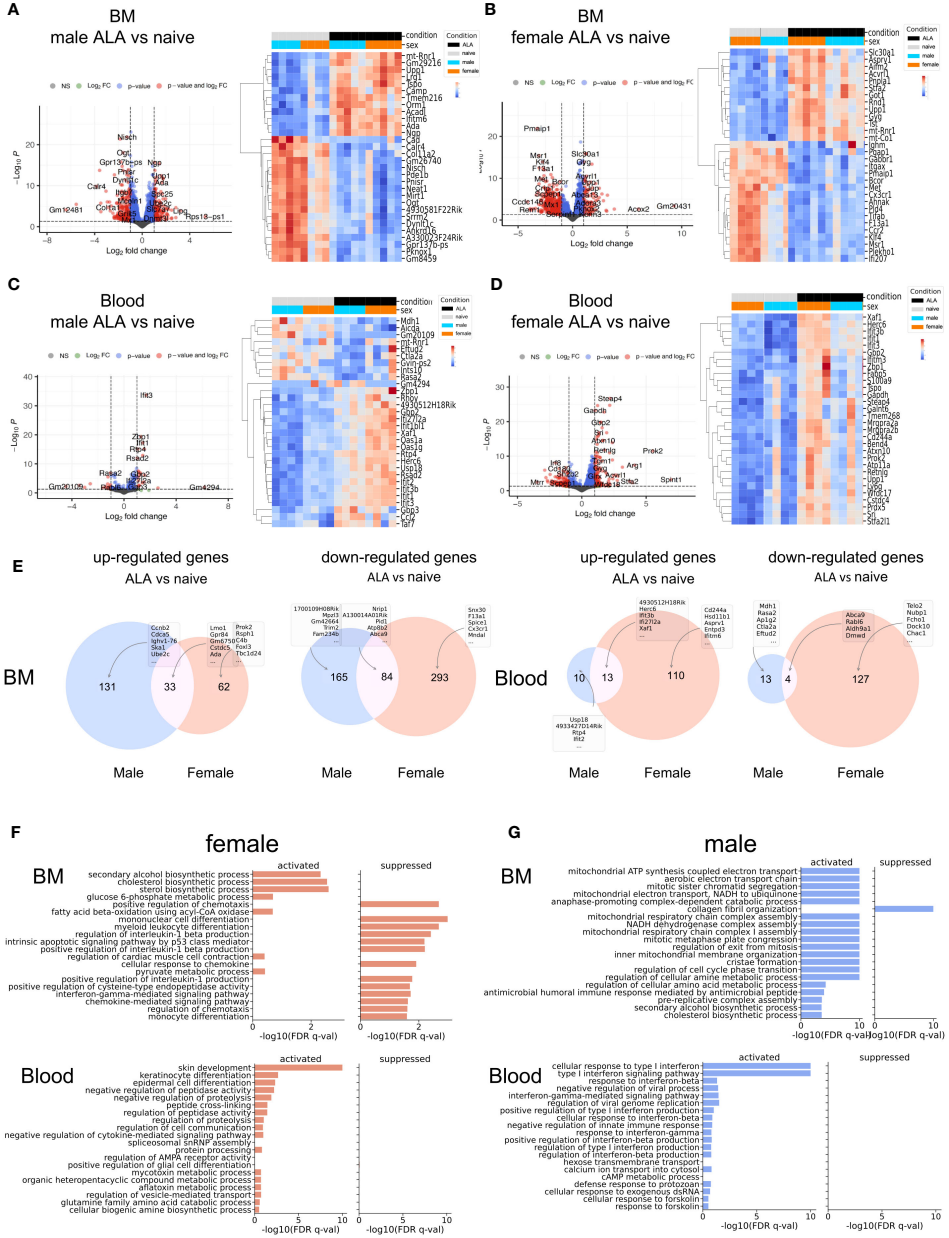
Transcription and Pathway analyses and maturity stage of BM- and blood neutrophils. Volcano plots show differential expression of genes in infected versus uninfected BM-derived **(A, B)** and blood-derived **(C, D)** neutrophils from male and female mice. X-axis: log2-fold change (FC); Y-axis: negative log padj of each gene. DESeq2 was used to calculate FC and padj (left side = low in infection, right side = high in infection). The Heatmap depicts the top differentially expressed genes from the Volcano Plot in both sexes. **(E)** Venn diagrams: for each condition and neutrophil origin. **(F, G)** Top 20 pathways showing the most significant differential changes in gene expression between ALA and WT mice for females (f) **(D)** and males (m) **(E)** Bar plots showing the z-scores for each pathway (activation versus suppression).

To assess sex-specific differences in neutrophils at the transcriptomic level, we conducted a differential expression analysis of neutrophils isolated from BM and blood samples from both male and female mice. Due to the extremely low number of neutrophils in the female liver (**Figure 1E**), we did not sufficient amounts of RNA required for high throughput sequencing. Our analysis revealed major differences in gene expression profiles during infection, with a focus on differentially expressed genes (DEGs) exhibiting a log-fold change (FC) > 1.5 and a p-adjusted < 0.01. The results are depicted in **Figures 3A–D**. In BM-derived neutrophils, we identified 413 DEGs in males and 472 DEGs in females during infection, when compared to uninfected controls. In blood-derived neutrophils, 40 DEGs were observed in males, and 254 DEGs were identified in females during infection (**Figures 3A-D**). A detailed list of all DEGs is provided in **Supplementary Table 4**. Infection-dependent differences in the expression of genes were more prominent in blood compared to BM-derived neutrophils from male and female mice. The differential changes of male ALA versus naïve and female ALA versus naïve show similar expression changes (**Figure 3A**). We visualized the normalized expression values of the DEGs in two heatmaps (**Figures 3A, B**, right side) for bone marrow to compare expression levels across conditions and sexes. Although the differences were not strongly pronounced, they were discernible in individual samples and between the sexes. For instance, a subcluster was observed in the female ALA versus naive comparison that was distinct from the male cluster, indicated in **Figure 3B**, that is not visible in the male comparison of BM (**Figure 3A**). In blood neutrophils, infection led to a more pronounced upregulation of genes, with females showing higher activation in the infected phase (**Figure 3D**) compared to expression changes in males (**Figure 3C**). Female blood neutrophils displayed upregulated expression of *Arg1* (FC = 4.11; padj < 0.01), a gene associated with N2-like neutrophils (**Figure 3B**) (30, 31). The heatmaps depict a rather homogenous distribution of gene expression levels throughout the samples. Further, Volcano plots stratified by sex illustrated lower expression in male ALA BM-derived neutrophils than in uninfected BM-derived neutrophils. Notably, sex differences were more pronounced in blood compared to BM-derived neutrophils from both male and female mice (**Supplementary Figures 4 A, B**). In Venn diagrams, we observed that a lower number of DEGs were down-regulated in male BM-derived neutrophils (249/413) compared to females (322/472) during infection. Conversely, more DEGs were upregulated in male neutrophils (164/413). In blood neutrophils, we found fewer downregulated DEGs in males (17/40) than in females (131/254), while more upregulated DEGs were observed in females (123/254) than in males (23/40) (**Figure 3E**).

We also investigated the infection-related enrichment of general signal transduction pathways and depicted the top 20 pathways of GO biological processes (GOBP). A detailed list of all GOBP is provided in **Supplementary Table 1**. These were separated into females (**Figure 3F**) and males (**Figure 3G**). In BM-derived neutrophils, infection was associated with the upregulation of pathways related to biosynthetic processes and increased cell proliferation. In contrast, blood neutrophils exhibited strong activation of pathways related to type I and type II interferon signaling. In summary, during infection, more genes, especially ISGs, were upregulated in blood neutrophils compared with BM-derived neutrophils, with greater activation of gene expression in female neutrophils (**Figures 3F, G**).

### Sex differences in the induction of type I and type II ISGs in neutrophils from the BM and blood

**Figure 4:**
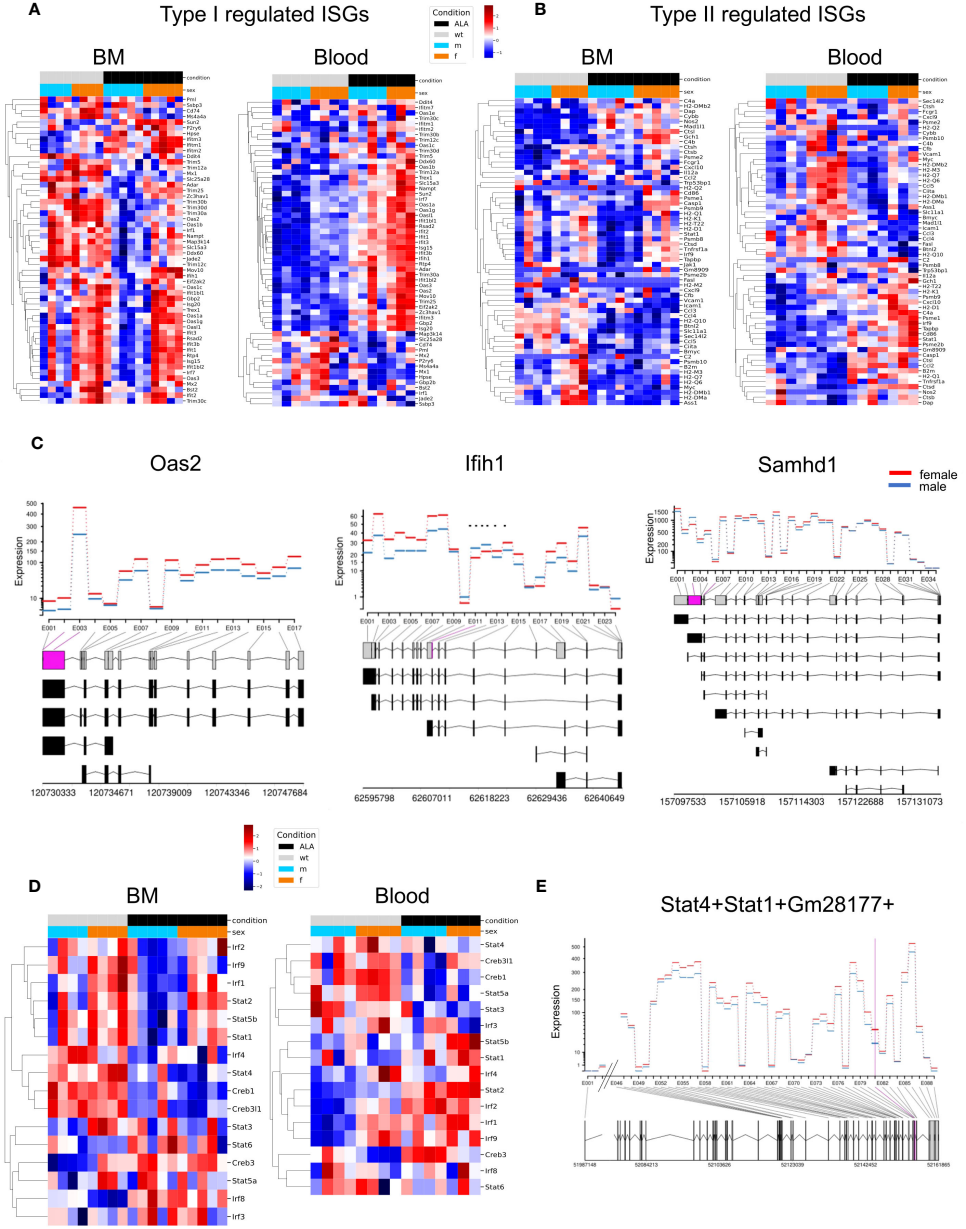
Sex differences in expression of type I- and type II-associated interferon-stimulated genes (ISGs). Heatmaps showing regulation of type I **(A)** - and type II **(B)** regulated ISGs in bone marrow (BM) and blood-derived neutrophils. Samples are grouped by condition (naive or ALA) and sex (male or female); red = up-, blue = downregulated. **(C)** DEU plots comparing gene expression in bone marrow neutrophils from naive female and male mice from selected ISGs. Significantly different exon transcripts (padj ≤ 0.05) are shown in purple. **(D)** Heatmaps showing regulation of transcription factors (TFs) in neutrophil from BM and blood. Samples are grouped by condition (naive or ALA) and sex; red = upregulation, blue = downregulation). **(E)** DEU plot for the TFs Stat1 and Stat4. Comparison between sexes neutrophils from naive BM.

Since interferon signaling is important for pathogen elimination, we next focused on sex- and infection-dependent expression of type I and type II ISGs in BM and Blood neutrophils (16, 31) (included ISGs are shown in **Supplementary Table 5**). Overall, female BM-derived neutrophils showed higher expression of type I ISGs under steady state conditions (BM and blood, *Oas2, Ifih1, Rsad2*), whereas a subset of ISGs was also highly regulated in their male counterparts (BM, *Ddit4, Slc25a28, Trim30b, Irf1*, *Jade 2;* Blood*, Ssbp3*) (**Figure 4A**). During infection, more ISGs were upregulated in female (comparison ALA *vs* naive) blood neutrophils than in male samples (comparison ALA *vs*. naive), whereas a downregulation of ISGs was observed in male neutrophils and no changes were found in female BM neutrophils (**Figure 4A**). Type II ISGs in BM neutrophils showed less pronounced sex differences under steady state conditions, however, expression of chemokines involved in adhesion and recruitment of neutrophils was higher in male-derived neutrophils. In blood neutrophils, in the steady state (e.g. *Myc, H2-DMb2, H2-M3, H2-Q7*…) but also during infection e.g. *Cxcl10, H2-D1, C4a, Psme1, Stat1, Psme2*, more genes were upregulated in neutrophils from female mice. However, one set of type II ISGs was also upregulated in male-derived neutrophils following infection e.g. *Casp1, CtsI, Ccl2* (**Figure 4B**). An additional illustration, showing organ-and sex-specific comparative analysis of ISGs, is provided in **Supplementary Figures 4C, D**.

Differential exon usage (DEU) is a type of alternative splicing that involves differential inclusion or exclusion of one or more exons from the mature mRNA transcript of a gene. The differential exon usage is calculated comparing infected versus naïve samples for males and females separately (**Figures 4C, E**). The figures show the fitted expression values (model effect estimates) of the comparisons for each of the exons of the genes for male (blue) and female (red).

We obtained 90 (female) and 1 (male) DEU for blood female and male, respectively, while female and male BM had 42 and 3 DEU, respectively (**Supplementary Table 2**). The three ISGs (*Oas2*, *Ifih1*, and *Samhd1*) showed a higher fitted expression of exons in females than in males BM neutrophils (**Figure 4C**). Interestingly, the number of DEUs was higher in BM- and blood neutrophils from females than males, suggesting a more versatile rearrangement and reaction to infection in females.

Furthermore, we aimed to gain insight into the transcription factor changes that might explain differential gene regulation in sex-specific transcriptional profiles in blood and BM neutrophils. We employed transcription factor interference to analyze the activity profiles of ISG-related transcription factors (TFs) and identified differentially activated TFs (**Supplementary Table 3**). We found that *Irf2*, *Irf1*, and *Stat2* exhibited female-specific upregulation in infected BM, while their expression was upregulated in blood of both, male and female, under disease conditions. *Creb3* was downregulated in uninfected female blood samples and downregulated for uninfected male samples of BM (**Figure 4D**). Additionally, a higher activity of *Stat1* and *Stat4* in female BM-derived neutrophils at steady state could be detected. This was verified by a higher *Stat1/Stat4* exon expression in female BM neutrophils under steady state conditions (only the part that changes significantly are shown) (**Figure 4E**).

In conclusion, type I ISGs are more highly expressed in female neutrophils in the blood, independent of infection. The expression of type II ISGs of neutrophils in blood is higher in female than in those of male individuals both in the naive state and after infection. Furthermore, the high number of DEUs in BM- and blood neutrophils from females suggest a more versatile rearrangement and reaction to infection in females.

### Sex difference in viperin/RSAD2 protein production by neutrophils

**Figure 5:**
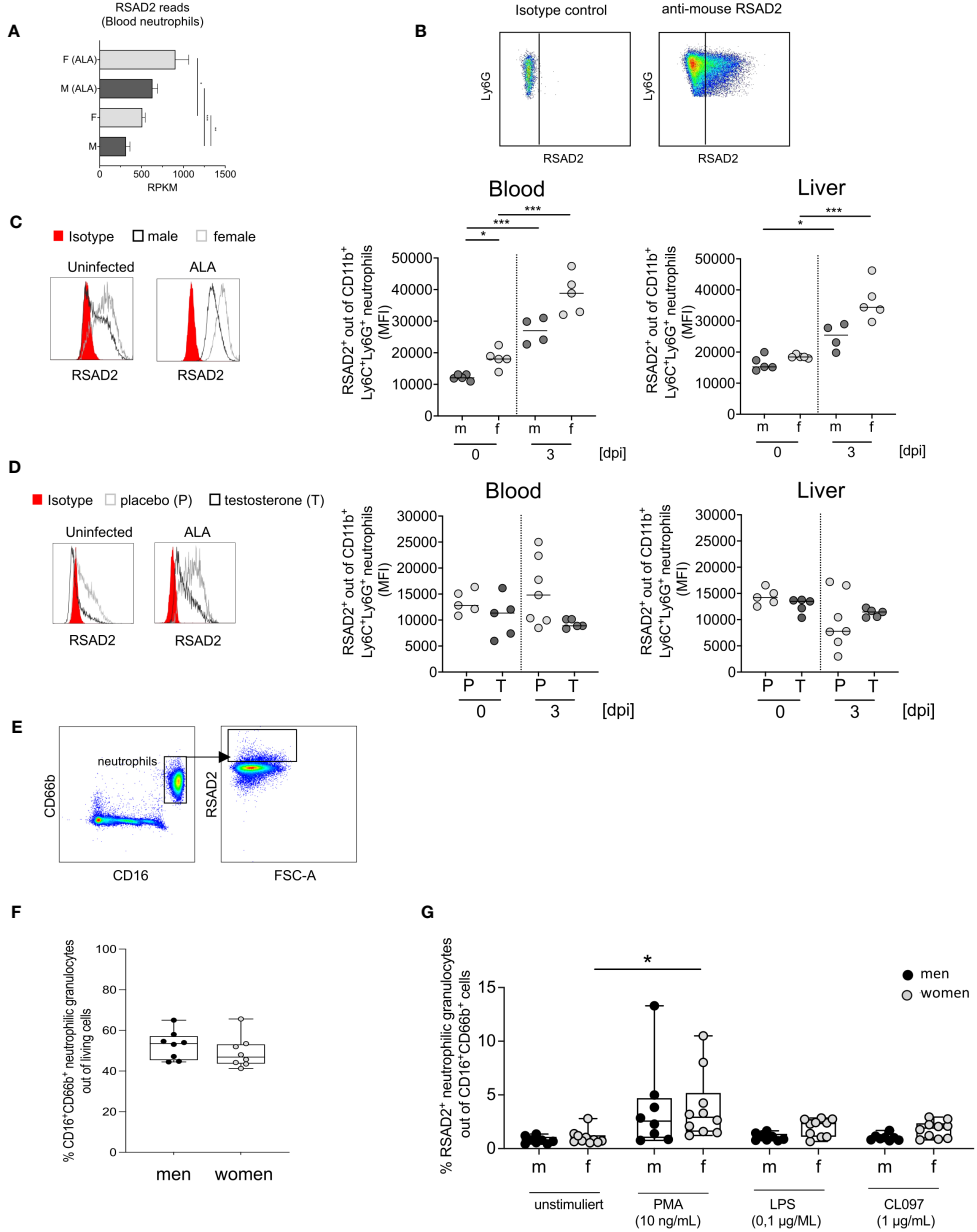
Expression of RSAD2 by neutrophils from mice and men. **(A)** RSAD2-Reads per Kilobase Million (RPKM) from murine blood of naive (F+M) and infected (amebic liver abscess/ALA) neutrophils. **(B)** Isotype and RSAD2 intracellular staining of blood and liver -derived neutrophil. **(C)** Histogram of RSAD2-expressing neutrophils from male and female mice and MFI of RSAD2^+^ expression in neutrophils from blood and liver and **(D)** Histogram of RSAD2-expressing neutrophils from placebo (P) and testosterone (T) substituted female mice (0 and 3 days post infection [dpi]). **(E)** Gating strategy to identify human neutrophils (CD66b^+^CD16^+^) expressing RSAD2 following stimulation. **(F)** Percentage of human neutrophils in men and women in peripheral blood. **(G)** Percentage of RSAD2^+^ from blood-derived human neutrophils following 4 hours incubation with different stimulants. **(C, D)** *p*-values were calculated using a two-tailed Student’s t test (*p < 0.05, **p < 0.01, ***p < 0.001). **(A, F, G)**. Mann-Whitney U Test *p < 0,05 and **(F, G)** Wilcoxon-Rank-Sum-test (*p < 0.05; **p < 0.01).

We have observed increased expression of type I ISGs at the transcriptional level, we were now interested in studying the expression and testosterone dependence of a prototypical ISG. For this purpose, we chose one of the best-studied ISGs, the viperin/RSAD2 (**Figure 4A**; **Figure 5A**) (32–34) and analyzed it by flow cytometry (**Figure 5B**). Upon infection, RSAD2-expressing neutrophils significantly increased in both sexes in the blood (***p < 0.001) and the liver (*p< 0.05 (males); ***p < 0.001 (females)) (**Figure 5C**). Following testosterone substitution of female mice, a noticeable trend towards decreased RSAD2-expressing neutrophils from blood, but not the liver, was observed after infection (**Figure 5D**). Unlike in mice, we found no sex difference in the percentage of peripheral neutrophils (FACS Plot of human neutrophils expressing RSAD2, **Figure 5E**) between men and women (**Figure 5F**), however, RSAD2 expression by neutrophils from women was significantly higher after stimulation with PMA compared to unstimulated neutrophils (*p<0.05, **Figure 5G**). After stimulation with LPS or CL097, a tendency for higher RSAD2 production was observed in female neutrophils compared with male neutrophils (**Figure 5G**).

In conclusion, male neutrophil granulocytes exhibit lower type I ISG RSAD2 expression compared to females, which is likely influenced by testosterone.

## Discussion

In the present study, we focused on sex differences and the effect of testosterone on neutrophil granulocytes in a mouse model of hepatic amebiasis. The first striking results became apparent when we compared the percentages of neutrophils in male and female mice in different organs. Already in naïve mice, we observed higher neutrophil amounts in the blood of males than in females without apparent difference in BM and liver. Following intrahepatic infection, this sex-related difference in neutrophil numbers was present in all organs. Previous investigations showed that testosterone modulates accumulation and recruitment of neutrophils, independent of infection-associated stimuli or site of infection (46–48), a finding that we were able to verify in this study. In the context of human studies, a higher count of neutrophil granulocytes was observed in the blood of males compared to females under healthy conditions, which aligns with the results obtained from the mouse experiments. However, within the same study, a greater percentage of neutrophils was identified in the blood of females. Notably, this proportion exhibited an age-dependent decline, becoming markedly reduced around the age of 50 years (49). An explanation for this phenomenon can be derived from another study demonstrating an anti-apoptotic influence of estrogens on neutrophils (50). The androgen receptor (AR), located on the x chromosome, is broadly expressed by all neutrophil lineages (51) and it has been shown that androgens not only stimulate proliferation of neutrophils and their precursors *in vitro* (52), but also accelerate recovery of neutrophil numbers *in vivo* following immunosuppressive therapies (53, 54) and more recently in a testosterone dose-response study in men (55). The observed increase in neutrophil frequencies in female mice following testosterone substitution in this study supports this direct effect of testosterone.

Since parasite growth in the liver is controlled more quickly in female mice (22), the higher number of neutrophils in males could be attributed to prolonged inflammatory processes due to parasite persistence and a general, androgen-dependent immunosuppressive effect on neutrophils. Increased expression of CXCL1 by neutrophils during ALA may support the hypothesis of inefficient initial infection control in males, leading to further excessive recruitment of neutrophils. Moreover, the anti-infection activity of these recruited neutrophils is presumably hampered because androgens modulate a variety of immune regulatory functions, including degranulation and ROS production, as well as promoting production of anti-inflammatory cytokines such as TGF-β1 or IL-10 (48, 56, 57).

Over the past years, studies show that neutrophils possess greater functional diversity than previously assumed. In an attempt to better estimate sex-specific and infection-specific differences in neutrophils, we analyzed the neutrophils according to the proinflammatory N1-like and anti-inflammatory N2 phenotype (19, 20). Unlike in the tumor environment, immature neutrophils (N0) expressing CD117 accounted for only a very small proportion of total neutrophils (<5%), mainly in bone marrow but also in blood and liver of naive animals, and were therefore not examined in detail in this study. The greatest increase in the percentage of N1- and N2-like neutrophils was found in the blood and liver of infected female mice and in the liver of infected male mice, indicating that neutrophils were most specifically polarized in the liver. Depletion of androgens by gonadectomy reduced the number of immature neutrophils in the BM, and testosterone reduced N1/N2 polarization of neutrophils in the blood, both before and during infection (compared with gonadectomized mice). Deconvolution analysis provided additional insight into the maturity state of neutrophils according to the nomenclature of Xie (17). The more premature phenotypes of neutrophil granulocytes (G2, G3, and G4) are found primarily in bone marrow. Compared to BM neutrophils, blood neutrophils have a high proportion of mature G5a, G5b, and G5c phenotypes, an observation also confirmed by Xie et al. It has been described that the G5b maturation state has a high ISG signature (17), which supports the results and data we obtained in this work. Furthermore, we examined the transcriptome of neutrophils from infected male and female mice, and focused on sex differences in type I and type II interferon (IFN) pathways. Type I IFNs (IFN-α, IFN-β, IFN-ϵ, IFN-κ) play important roles in neutrophil biology by signaling via a common receptor interferon-α/β receptor (IFNAR), thereby inducing expression of several hundred ISGs (58). These IFNs regulate the oxidative burst and formation of extracellular traps, enhance production of IL-6 and *TNF* mRNA in male human neutrophils upon TLR7/8 stimulation, despite similar expression of their receptor in male and females (16, 59, 60). By comparing induction of ISGs, we found striking sex differences between neutrophils from the BM and blood. In the blood under steady state conditions, upregulation of type I ISGs was higher in neutrophils from females than in males. This difference was more pronounced following infection. In blood neutrophils, type I ISGs were more upregulated in females compared to males following infection, suggesting a less activated phenotype in males.

In humans, greater upregulation of type I ISGs was observed in neutrophils from healthy adult women compared with men including expression of RSAD2 on the mRNA level (16). Here we found that RSAD2 expression on the protein level is also higher in women compared to men and downregulated by testosterone in mice. Some studies have indicated that Viperin expression can be induced in response to parasitic infections, suggesting that it might have a broader antiparasitic role beyond its well-established antiviral function. For example, research has shown increased RSAD2 expression in response to infections caused by protozoan parasites like *Toxoplasma gondii* (61) and *Plasmodium falciparum* (62), respectively. However, the specific interactions between RSAD2 and parasites and the extent of its influence on parasitic infections require further investigation.

By contrast, regulation of type II ISGs (30), is less clearly associated with infection, and essentially two groups were identified in blood neutrophils: one in which genes are upregulated under steady state conditions and downregulated in the infectious state and a second in which the opposite occurred.

One ISG group of interest is associated with leukocyte migration; these include IP-10, CCL2, and CCL3. The levels of these chemokines in blood serum were analyzed in the present study, in addition to transcriptional analysis. These molecules are known chemoattractants for monocytes (63, 64) and neutrophils (23) and are present in the type II ISG heatmaps.

In summary, we found sex-specific differences in neutrophils, including recruitment behavior, maturation stage, and variations in transcription factors and ISGs like RSAD2, which are partly influenced by androgens. The less activated and mature state of male murine neutrophils results in prolonged survival of amoebic trophozoites, leading to a deleterious cycle that contributes to liver destruction.

## Data availability statement

The datasets presented in this study can be found in online repositories. The names of the repository/repositories and accession number(s) can be found below: GSE242045 (GEO).

## Ethics statement

The studies involving humans were approved by Medical association Hamburg (permission number: 2020-10067-BO). The studies were conducted in accordance with the local legislation and institutional requirements. The participants provided their written informed consent to participate in this study. The animal studies were approved by Federal health authorities of the State of Hamburg (permission numbers: N51/17; N120/2020). The studies were conducted in accordance with the local legislation and institutional requirements. Written informed consent was obtained from the owners for the participation of their animals in this study.

## Author contributions

ME: Data curation, Formal Analysis, Investigation, Methodology, Software, Supervision, Validation, Visualization, Writing – review & editing. SH: Data curation, Formal Analysis, Methodology, Software, Supervision, Writing – review & editing. MR: Data curation, Investigation, Methodology, Validation, Visualization, Writing – review & editing. DM: Formal Analysis, Investigation, Methodology, Validation, Writing – review & editing. FH: Data curation, Formal Analysis, Methodology, Software, Validation, Visualization, Writing – review & editing. RK: Data curation, Formal Analysis, Methodology, Software, Validation, Visualization, Writing – review & editing. CH: Formal Analysis, Investigation, Methodology, Validation, Writing – review & editing. JB: Formal Analysis, Investigation, Methodology, Writing – review & editing. VB: Formal Analysis, Methodology, Validation, Writing – review & editing. BH: Methodology, Supervision, Validation, Writing – review & editing. AB: Methodology, Formal Analysis; Validation, review & editing. MG: Investigation, Methodology, Validation, Writing – review & editing. HF: Investigation, Methodology, Supervision, Writing – review & editing. CM: Methodology, Visualization, Writing – review & editing. DC: Data curation, Formal Analysis, Methodology, Software, Validation, Writing – review & editing. SB: Conceptualization, Data curation, Formal Analysis, Funding acquisition, Resources, Software, Supervision, Validation, Writing – review & editing. JS: Conceptualization, Data curation, Formal Analysis, Investigation, Methodology, Supervision, Validation, Visualization, Writing – review & editing. HL: Conceptualization, Data curation, Funding acquisition, Investigation, Project administration, Resources, Supervision, Visualization, Writing – original draft, Writing – review & editing.
