# Supplementary material for: Testosterone affects type I/type II interferon response of neutrophils during hepatic amebiasis
Source: Front Immunol. 2023 Dec 21;14:1279245. doi: 10.3389/fimmu.2023.1279245 (PMC10764495; doi:10.3389/fimmu.2023.1279245)
Supplement: Supplementary file 1 [file DataSheet_1.pdf]

Male sex affects type I/type II interferon response of neutrophils during hepatic amebiasis

Marco-Er-Lukowiak[#1], Sonja Hänzelmann[#2 3 4], Moritz Rothe[1], David Teodor Moamenpour[1], Fabian Hausmann[2 3], Robin Khatri[2 3], Charlotte Hansen[1], Jennifer Boldt[1], Valentin Andreas Bärreiter[1], Barbara Honecker[1], Annika Bea[1], Marie Groneberg[1], Helena Fehling[1], Claudia Marggraff[1], Dániel Cadar[1], Stefan Bonn[3,4,5], Julie Sellau[†1], and Hanna Lotter[†1]

Table of contents

Supplementary materials and methods

Supplementary Fig. 1

Supplementary Fig. 2

Supplementary Fig. 3

Supplementary Fig. 4

References

Supplementary materials and methods

Neutrophil isolation for functional assays

Neutrophil granulocytes from bone marrow (BM) or from peripheral organs (spleen and blood) were isolated using the Neutrophil Isolation Kit from Miltenyi (#130-097-658, Miltenyi Biotec) before they were introduced in the different *in vitro* assays. Splenocytes and blood leukocytes were obtained as previously described but under sterile conditions.

Determination of neutrophil phagocytic activity

Neutrophil phagocytic capacity was performed using the Phagocytosis Assay Kit (IgG FITC) (#500290, Cayman chemical). $1*10^5$ isolated Neutrophil granulocytes from bone marrow (BM), $3*10^5$ blood leukocytes and $1*10^6$ splenocytes were co-incubated with FITC-labeled IgG beads at 4 °C or 37 °C for 1 hour. Cells were then prepared according to the kit instructions, stained with fluorochrome attached antibodies (CD11b FITC (1:200, M1/70), CD11b PerCP (1:100 M1/70), Ly6C PerCP/Cy5.5 (1:400, HK 1-4) and Ly6G PE (1:400, 1A8)) and quantified by flow cytometry.

Determination of Reactive oxygen species (ROS)

Neutrophils were washed with ROS buffer (RPMI medium, 1% BSA, 1 mM $CaCl_2$), counted, and diluted to 10^6 cells/mL. 10^5 Neutrophils were mixed with ROS detection reagent dihydrorhodamine 123 (500 µg/ml) and incubated at 37 °C for 15 min. Phorbol-12-myristate-13-acetate (PMA) was added at two different concentrations (5 nM; 10 nM) and incubation was continued for another 45 minutes. Cells from splenocyte suspension were stained with fluorochrome labeled antibodies. ROS production was quantified using the FITC channel of a flow cytometer.

Determination of neutrophil degranulation (MPO ELISA)

Degranulation assay was performed with purified neutrophils derived from the bone marrow and periphery. In brief, 10^5 neutrophils were incubated with Phorbol12-myristat-13-acetat (PMA) (100 nM) at 37° C for 2 hours. After incubation neutrophils were centrifuged (300 x *g*, 4 °C, 5 min) and the supernatant collected and stored at -20 °C. Supernatants were analyzed using a murine ELISA for the detection of myeloperoxidase (MPO) (R&D Systems).

In vitro stimulation of purified neutrophils

Isolated neutrophil granulocytes from bone marrow were placed in a 96-well round-bottomed plate, so that each well contained $1x10^6$ cells. The plate was centrifuged (350 x *g*, 4 °C, 5 min) and the supernatant was discarded. Neutrophils were resuspended in 200 µL of cRPMI

containing the appropriate stimulus reagent (*Entamoeba histolytica* antigen (E. his Ag) 1 mg/mL, Phorbol12-myristat-13-aceta (PMA) 10 ng/ml, lipopolysaccharide (LPS) 5 µg/mL, 2-(Ethoxymethyl)-3H-imidazo(4,5-c) quinolin-4-amine (CL097) 1 µg/mL and N-Formyl-Met-Leu-Phe fMLP 5 µM). Neutrophils were incubated at 37 °C and 5% CO_2 for 4 hours. Supernatants were collected and stored at -20 °C while neutrophils were stained for flow cytometry.

Preparation of E. histolytica lysate

Trophozoites from the pathogenic B2 *E. histolytica* clone were harvested and centrifuged at 250 x *g* for 4 °C, 5 minutes. The supernatant was discarded and the pellet was frozen and thawed three times using liquid nitrogen. Protein concentration was measured using the Nanodrop^{TM} 2000 spectrometer (Thermo Scientific).

Cytokine analysis

Stimulated neutrophil supernatant was used for cytokine analysis. The collected blood was centrifugated (1000 x *g*, 4 °C, 10 min) to obtain the plasma and stored afterwards -20 °C for cytokine measurements. Cytokine analysis was performed using multiple customized, murine LEGENDplex kits (Biolegend) for TNF-α, IL-1β, CXCL1, CXCL10, GM-CSF, IL-10, IL-4, IL-6, IL-13, IL-23, CCL2, CCL3, IL-17A, IL12p70, IFN-γ and IFN-β.

NO_2^- analysis

For the measurement of NO_2^- concentrations in the blood, plasma was incubated with Griess Reagent (Promega Corporation). The assay was performed on a 96-well flat bottom plate, in which six 1:2 dilutions of a NO_2^- standard (100 µM) were provided. Serum samples were diluted 1:3 followed by the addition of sulfanilamide solution to each standard and sample well. After 7 minutes of incubation, N-(1-Naphthyl)ethylendiamin (NED) solution was added to all wells. After 7 minutes, the change of color was detected using an ELISA-Reader (Dynex Technologies, Inc.) with the light absorption at 530 nm wave length. NO_2^- concentration correlated with the amount of absorbed light.

Supplementary figures

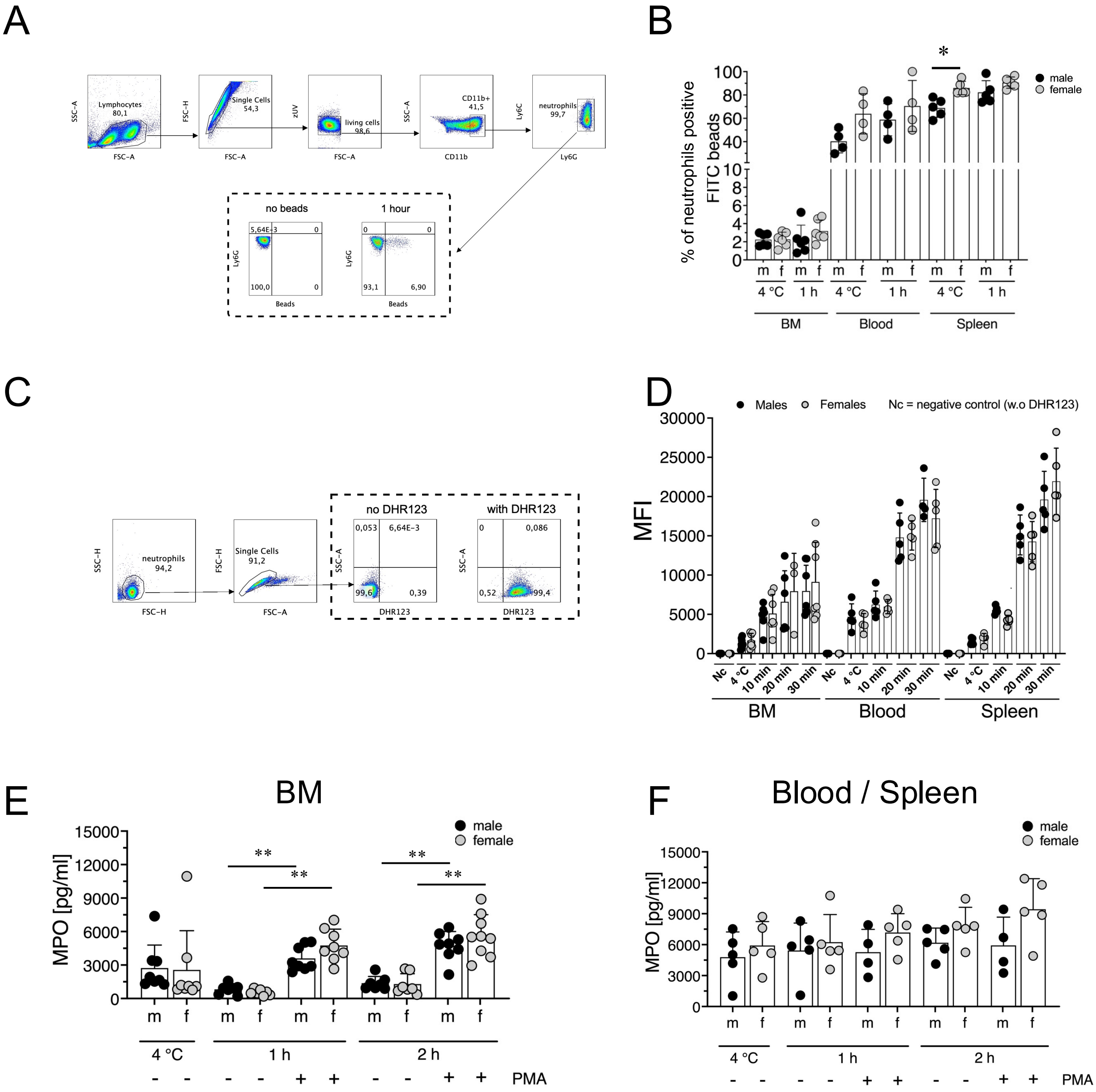

Suppl. Fig. 1. Sex- dependent analysis of phagocytosis, production of reactive oxygen species (ROS) and degranulation capacity of neutrophils. A Gating strategy to identify FITC$^+$ IgG labeled beads in neutrophils (CD11b$^+$Ly6C$^+$Ly6G$^+$) by flow cytometry. **B** Percentage of phagocytosed beads by isolated bone marrow neutrophils, neutrophils of blood leukocytes and neutrophils of splenocytes. Neutrophils were incubated at indicated temperatures and time points with fluorescent beads (1:400) and analyzed by flow cytometry. Comparison is shown between male (m) and female (f) mice (n=5-6). **C** Gating strategy for Dihydrorhodamin 123 (DHR123) identification in neutrophil granulocytes (CD11b$^+$Ly6C$^+$Ly6G$^+$) by flow cytometry. **D** Mean fluorescence intensity (MFI) of DHR123 in neutrophils isolated from BM, blood leukocytes or splenocytes. Neutrophils were incubated at indicated temperatures and time points with DHR123 (0,5 mg/ml) and analyzed by flow cytometry (n=4-7). **E-F** Myeloperoxidase (MPO) concentration (pg/ml) in supernatants of PMA (100 nM) stimulated neutrophils was analyzed with ELISA. The assay was performed with neutrophils isolated from bone marrow (**E**) and pooled spleen and blood (**F**) (n= 5-8). *P*-values were calculated using two-tailed Mann-Whitney-U test. (*$p < 0.05$, **$p < 0.01$).

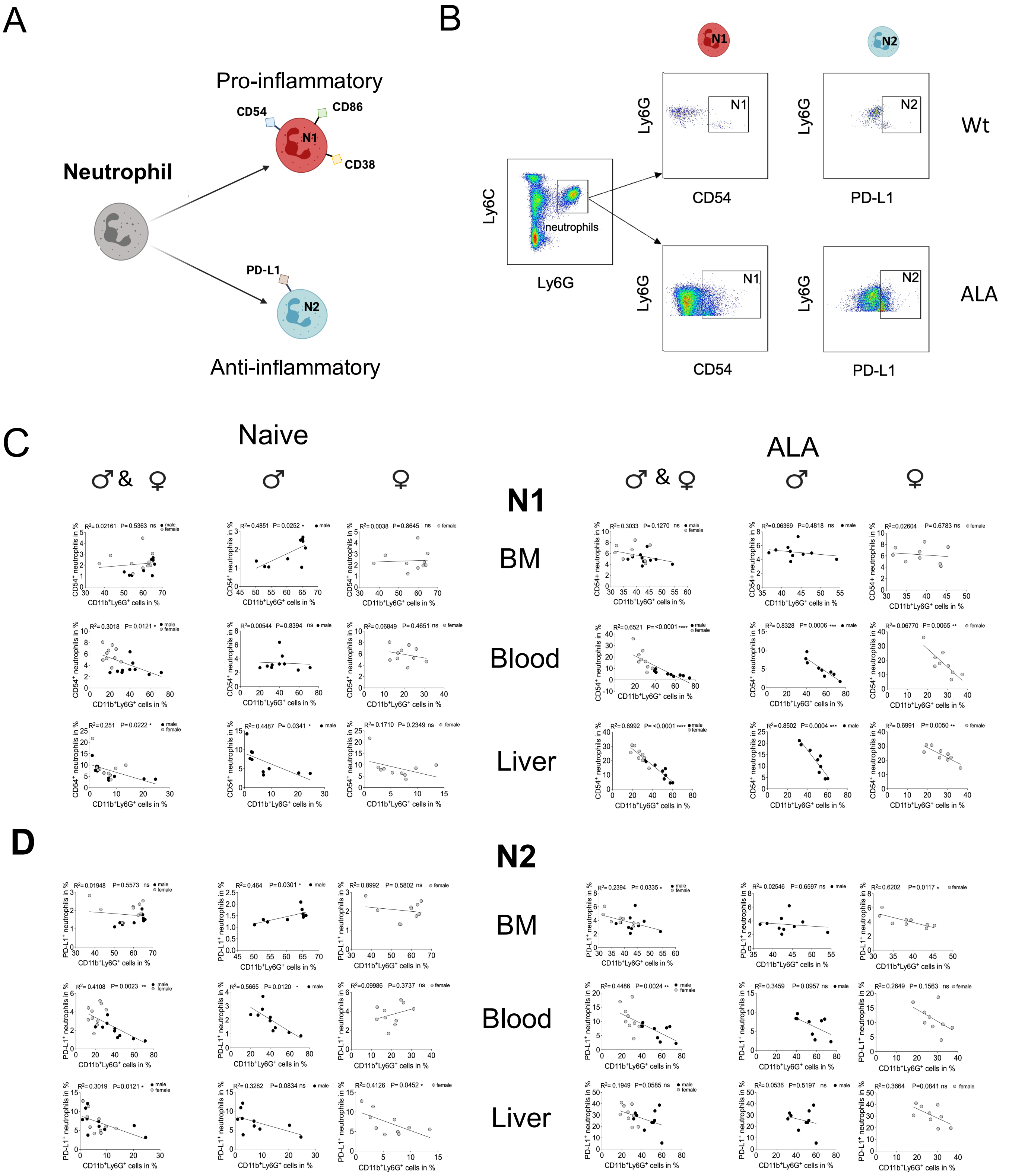

Suppl. Fig. 2 Neutrophil granulocytes polarize more strongly into N1 and N2 phenotypes in female mice compared to male mice during ALA. A. Scheme showing the neutrophil nomenclature suggested by Jaillon et al. 2020**B.** Representative flow cytometry plots showing the percentages of liver-specific N1 (pro-inflammatory; CD54), and N2 (anti-inflammatory; PD-L1) neutrophils on Day 3 post infection with *E. histolytica*. Organ-dependent correlation of pro-inflammatory N1 (**C**) and anti-inflammatory N2 (**D**) neutrophils from female and male mice. Correlations were calculated with both sexes included or separately. Analysis was performed with neutrophils derived from bone marrow (BM), blood and liver. Plots are shown for naïve conditions and on day 3 post infection. Correlation was calculated using two-tailed Pearson correlation coefficent (ns = non-significant; **P* < 0.05; ***P* < 0.01; ****P* < 0.001; *****P* < 0.0001).

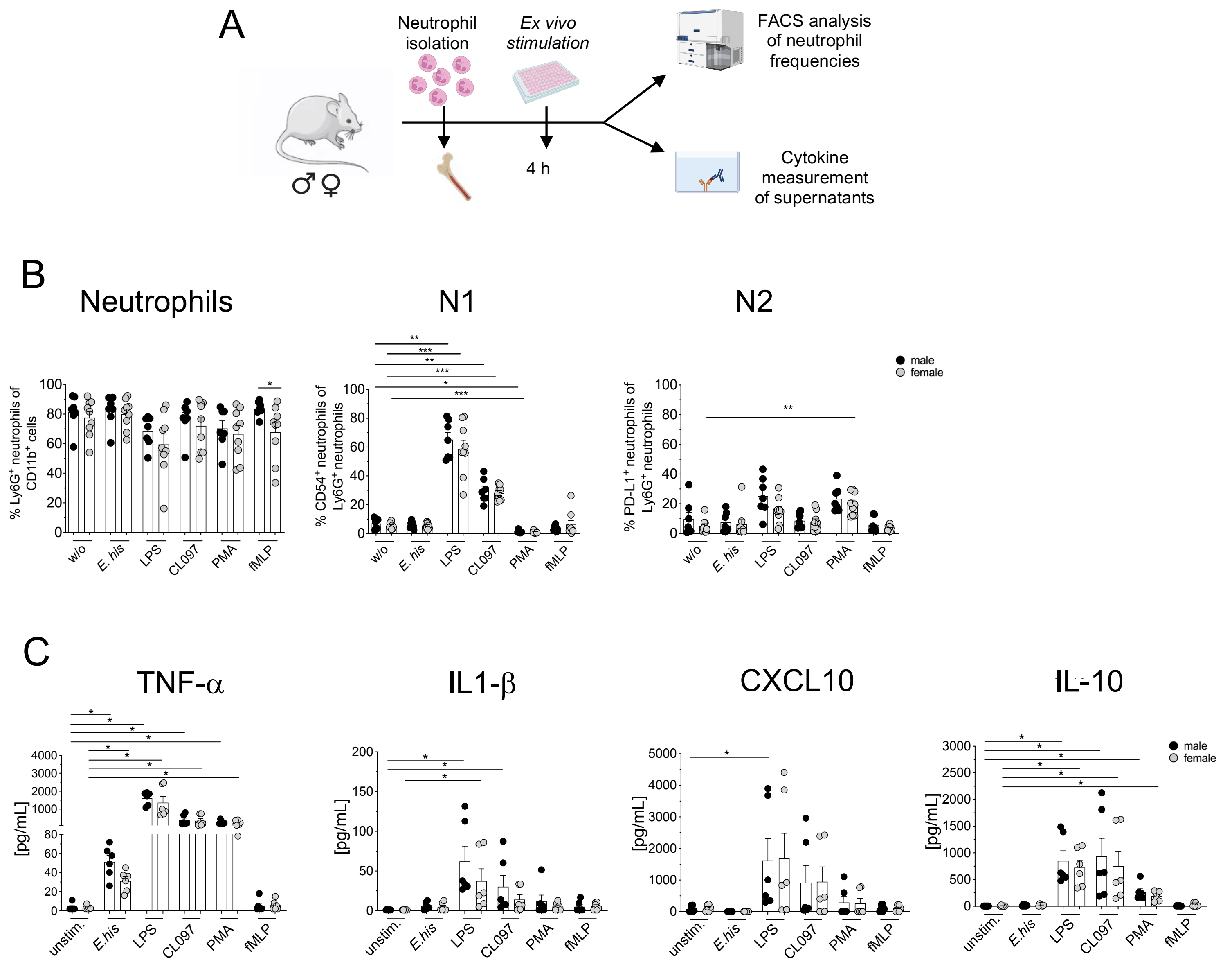

Suppl. Fig. 3 Influence of *ex vivo* stimulation on N1 and N2 polarization and related cytokines in BM-neutrophils. A Study design for *ex vivo* stimulation. Neutrophils were isolated from the bone marrow of male (m) and female (f) mice and stimulated for 4 hours with *E. histolytica* lysate (1 mg/ml), LPS (5 µg/ml), CL097 (1 µg/ml), PMA (10 ng/ml) or fMLP (5 µM) and analyzed by flow cytometry and cytokines were determined in the supernatant using ELISA or multicytokine bead assay. **B** Percentage of Ly6G$^+$ neutrophils out of CD11b$^+$ cells, CD54$^+$ neutrophils (N1) and PD-L1$^+$ neutrophils (N2) after stimulation with indicated reagents (n=7-9). **C** Concentrations (pg/ml) of N1 and N2 related cytokines in supernatants of neutrophils after stimulation with indicated reagents (n=5-6). *P*-values were calculated using Mann-Whitney U-Test (**p* < 0.05, ***p* < 0,01, ****p* < 0,001)

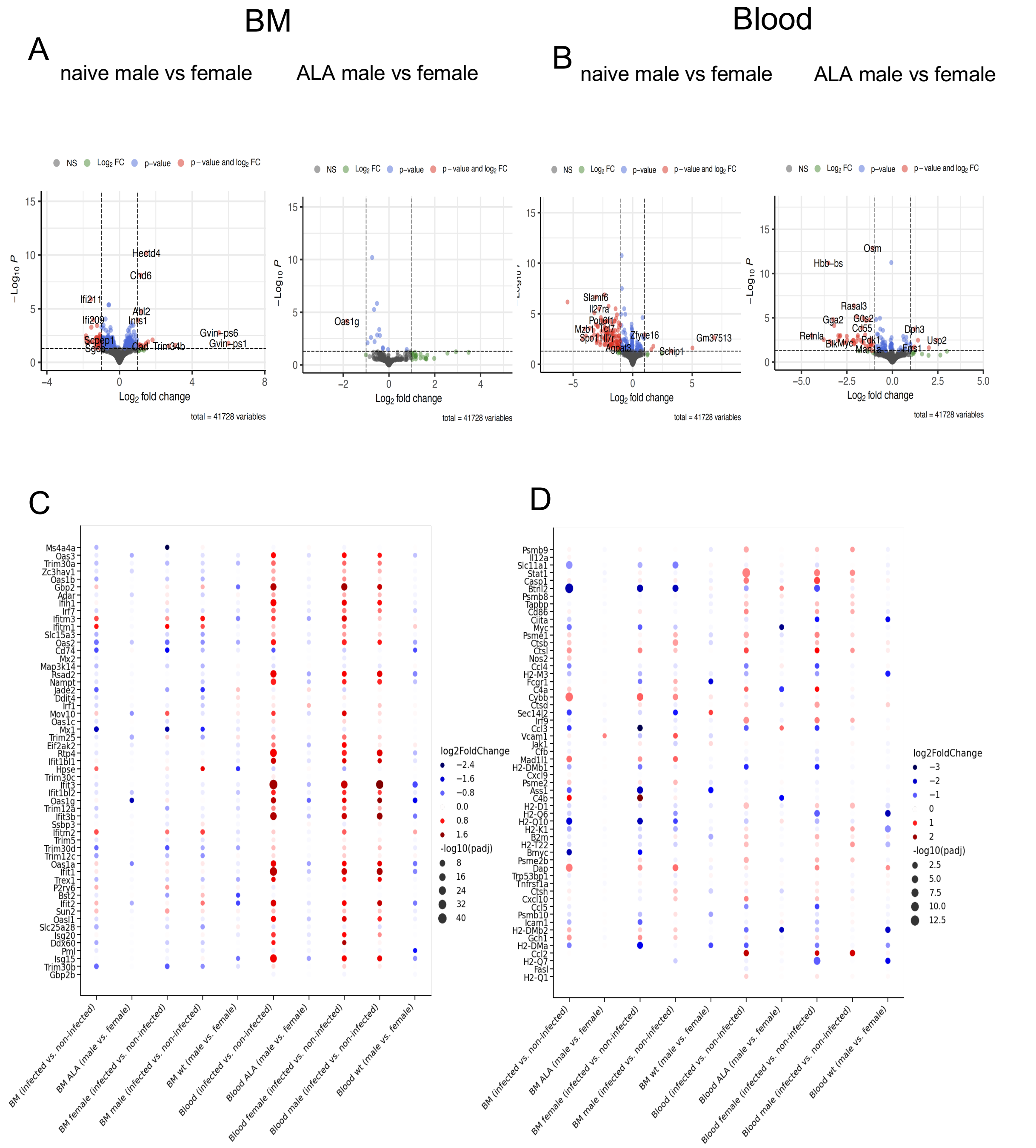

Suppl. Fig. 4 DEG and comparative gene analysis between sexes in BM and blood neutrophils Volcano plots show differential expression of genes in male vs female in BM (**A**) and blood neutrophils (**B**). X-axis: FC; Y-axis: negative log p-adjusted (padj) of each gene. DESeq2 was used to calculate log2 fold change and padj. Red circles indicate significant differential change: left side = decrease (low in infection), right side = increase (high in infection). Blue circles represent significance of adjusted p-values only. **(C, D)** Comparative analysis of ISGs between BM and blood (y-axis). Fold changes (color) and adjusted *p*-values (size) of the different conditions depicted on the x-axis.
